# Targeted Exon Sequencing Successfully Discovers Rare Causative Genes and Clarifies the Molecular Epidemiology of Japanese Deafness Patients

**DOI:** 10.1371/journal.pone.0071381

**Published:** 2013-08-13

**Authors:** Maiko Miyagawa, Takehiko Naito, Shin-ya Nishio, Naoyuki Kamatani, Shin-ichi Usami

**Affiliations:** 1 Department of Otorhinolaryngology, Shinshu University School of Medicine, Asahi, Matsumoto, Japan; 2 Laboratory for Statistical Analysis, RIKEN, Kanagawa, Japan; Innsbruck Medical University, Austria

## Abstract

Target exon resequencing using Massively Parallel DNA Sequencing (MPS) is a new powerful strategy to discover causative genes in rare Mendelian disorders such as deafness. We attempted to identify genomic variations responsible for deafness by massive sequencing of the exons of 112 target candidate genes. By the analysis of 216randomly selected Japanese deafness patients (120 early-onset and 96 late-detected), who had already been evaluated for common genes/mutations by Invader assay and of which 48 had already been diagnosed, we efficiently identified causative mutations and/or mutation candidates in 57 genes. Approximately 86.6% (187/216) of the patients had at least one mutation. Of the 187 patients, in 69 the etiology of the hearing loss was completely explained. To determine which genes have the greatest impact on deafness etiology, the number of mutations was counted, showing that those in *GJB2* were exceptionally higher, followed by mutations in *SLC26A4, USH2A, GPR98, MYO15A, COL4A5* and *CDH23*. The present data suggested that targeted exon sequencing of selected genes using the MPS technology followed by the appropriate filtering algorithm will be able to identify rare responsible genes including new candidate genes for individual patients with deafness, and improve molecular diagnosis. In addition, using a large number of patients, the present study clarified the molecular epidemiology of deafness in Japanese. *GJB2* is the most prevalent causative gene, and the major (commonly found) gene mutations cause 30–40% of deafness while the remainder of hearing loss is the result of various rare genes/mutations that have been difficult to diagnose by the conventional one-by-one approach. In conclusion, target exon resequencing using MPS technology is a suitable method to discover common and rare causative genes for a highly heterogeneous monogenic disease like hearing loss.

## Introduction

Etiological studies have shown that approximately two-thirds of congenital/early-onset sensorineural hearing loss in developed countries is estimated to be due to genetic causes [1]. Deafness is an extremely heterogenous disorder and the involvement of nearly 60 distinct nonsyndromic deafness genes sometimes makes the precise diagnosis difficult. To clarify individual etiology in such heterogenous diseases, one-by-one gene screening based on conventional PCR-based direct sequencing of candidate genes has been developed, and currently *GJB2* has become the first to be screened, followed by several commonly encountered genes. As more comprehensive screening methods, micorarray-based screening [2], [3] and Invader assay-based screening [4], [5] have also been developed. Recent advances in exome sequencing using Massively Parallel DNA Sequencing (MPS) have revolutionized the elucidation of genetic defects causing monogenic disorders [6]–[8]. A number of papers regarding gene discovery and successful clinical application for identification of responsible genes for deafness using MPS have recently been published [9]–[17]. In this study, we have chosen 112 genes (including 54 known deafness causing genes, 22 known syndromic hearing loss causing genes and 36 possible candidate genes which expressed highly in the inner ear) and conducted genetic analysis to 1) confirm the potentiality of MPS -based genetic screening strategies for such a genetically heterogenous disease, and 2) clarify molecular epidemiology by identifying responsible/candidate genes in a large number of patients using MPS technology.

## Materials and Methods

### Subjects

Two hundred sixteen Japanese patients with bilateral sensorineural hearing loss from 33 ENT departments nationwide participated in the present study. With regard to onset age (the age of awareness), 120 patients had early-onset deafness (below 6 y.o.), and 96 had late-detected deafness. Thirty subjects were from autosomal dominant or mitochondrial inherited families (two or more generations affected); 98 subjects were from autosomal recessive families (parents with normal hearing and two or more affected siblings) or had sporadic deafness (also compatible with recessive inheritance or non-genetic hearing loss). Hearing loss was evaluated using pure-tone audiometry (PTA) classified by a pure-tone average over 500, 1000, 2000 and 4000 Hz in the better hearing ears. For children who could not undergo PTA, we used an average over 500, 1000, 2000 Hz in either auditory steady-stem response (ASSR) or conditioned oriented reflex audiometry (COR), or the response threshold (dB) from auditory brainstem response (ABR). Computed tomography (CT) scans were performed to check for congenital inner ear anomalies.

The patients had already been evaluated by conventional PCR-based one-by-one gene screening and Invader-based multi-gene screening [5], and 61 out of the 216 (45/120 prelingual, 16/96 postlingual) patients were already found to have *GJB2* (n = 38), *SLC26A4* (n = 15), or mitochondrial 1555 (n = 3) and 3243 (n = 5) mutations. We chose these patients because 1) they were “randomly” selected, and 2) they had already been screened by Invader assay and further fully sequenced by Sanger sequencing for the previously found common and frequent deafness causing genes i.e., *GJB2, SLC26A4, KCNQ4,* and *CDH23*. Therefore, we could simultaneously use these 216 samples for both diagnostic purposes and for verification. As a control for pathogeneity of each genomic variation, 72 Japanese samples were used in this study, because they were 1) ethnically similar, 2) had normal hearing evaluated by pure-tone audiometry, and 3) were collected from throughout the nation, and were able to undergo identical procedures. All subjects or next of kin, caretakers, or guardians on the behalf of the minors/children gave prior written informed consent for participation in the project, and the Shinshu University Ethical Committee as well as the respective Ethical Committees of the other participating institutions of the Deafness Gene Study Consortium (Hokkaido University, Hirosaki University, Iwate Medical University, Tohoku University, Yamagata University, Fukushima Medical University, Jichi Medical University, Gunma University, Nihon University, Nippon Medical School, Nippon Medical School Tama Nagayama Hospital, Jikei University, Toranomon Hospital, Kitasato University, Hamamatsu Medical University, Mie University, Shiga Medical Center for Children, Osaka Medical College, Hyogo College of Medicine, Kobe City Medical Center General Hospital, Wakayama Medical University, Okayama University, Yamaguchi University, Ehime University, Kyushu University, Fukuoka University, Nagasaki University, Kanda ENT Clinic, Miyazaki Medical College, Kagoshima University, Ryukyus University) approved the study.

### Targeted Enrichment and DNA Sequencing

One hundred twelve genes listed in **Table S1**, including 54 genes reported to be causative of non-syndromic hearing loss (Hereditary Hearing loss Homepage; http://hereditaryhearingloss.org/) and 22 reported to cause syndromic hearing loss were selected for sequencing. In hopes of finding novel causative genes, we added 36 genes that are highly expressed in the adult human inner ear by microarray analysis [18]. DNA from 12 patients was pooled and 3 µg of each pooled DNA was used as an input material for SureSelect target DNA enrichment (Agilent Technologies, Santa Clara, CA) and Illumina GAIIx sequencing (Illumina, San Diego, CA) according to the manufacturers’ procedures. Each genomic DNA pool was fragmented using the Covaris™ S2 System (Covaris, Woburn, MA) to about 200 bp fragment length. After fragmentation, DNA fragments were blunt-ended and phosphorylated at the 5′ end using a Paired End Genomic DNA Sample Prep Kit (Illumina) and successively, adeninylated at the 3′ end and ligated to pre-capture adaptor olligonucleotides containing SureSelect target DNA enrichment kit. After adaptor oligonucleotide ligation, pre-capture amplification was performed with Heraculase II Fusion DNA polymerase (Agilent Technologies). Between each step of sample preparation, DNA pools were purified with the Agencourt AMPure XP system (Beckman Coulter Genomics, Danvers, MA). The Capture library was designed with Agilent’s eArray homepage (http://earray.vhem.agilent.com/earray/). The bait cRNA library contained all exons of 112 genes. Exons of selected genes of all variants were selected from RefSeq and Ensembl databases using the University of California Santa Cruz table browser (http://genome.ucsc.edu/). Adaptor ligated and pre-amplicated samples were hybridized to the Capture cRNA library at 65°C for 24 hours with SureSelect Hybridization buffer and successively captured with Dynabeads MyOne Streptavidin T1 beads (Invitrogen) and washed with SureSelect Wash buffer. After target capture, selected product from pooled DNA was post-amplified with Heraculase II Fusion DNA polymerase and Illumina Multiplexing Sample Preparation Oligonucleotide Kit and then submitted to the massive parallel sequencing in a lane on a Illumina GAIIx genome platform (Illumina).

### Mapping and Filtering

**Figure 1:**
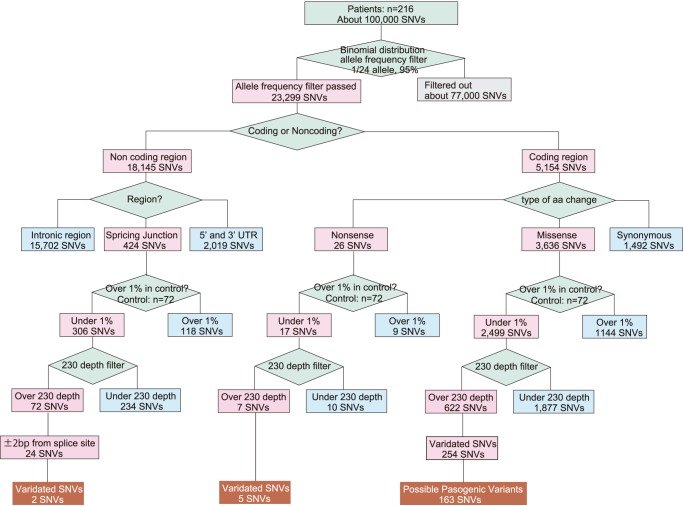
Algorithm applied in this study. **Nonsense mutations, splice-site mutations, and missense mutations were chosen according to this algorithm.**



The sequence data were processed with standard Illumina base calling procedure and successively mapped to human genome sequence (build hg 36) with the Bowtie program and BWA program [19], [20]. The two programs were used consecutively, because the Bowtie program cannot detect insertion/deletion efficiently. A total of 55.4 and 8.5 Gb sequences with about 9,000,000 and 1,400,000 reads were obtained by the pair-end method for the patients and the controls, respectively. After alignment, the filtering algorithm shown in **Fig. 1** was applied to collect the responsible genes/mutations. First, because of usage of pooled DNA samples, potential single nucleotide variants (SNVs) were filtered by the frequency of variant reads at each position. For the number of variants in each position, we assumed a binomial distribution with the probability parameter of 1/24, and the size parameter of the number of coverage. The largest integer number that is not larger than the value giving the cumulative distribution function of 0.025 of the binomial distribution was used as the threshold value, and the position was selected when the number of the reads of the variant were not lower than the threshold value indicated in formula (1).

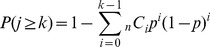
(1)In the formula, *n* denotes total depth (wild type+mutation allele) of each mapped position, *j* denotes the observed number of mutational alleles at each mapped position, and *p* denotes the relative frequency of the mutation allele in the pool. In this study DNA of 12 patients was pooled, and the minimal positive value of the relative frequency of the mutational allele in each pooled DNA sample should be 1/24. Therefore, we employed *p* = 1/24. To reduce false negative cases, we used *P* = 95% and after the calculation of this formula, *k* value indicated the number of minimal mutation allele copies that was used as the threshold for each mapped position. We fixed *p* = 1/24 and *P* = 95%, and then, *k* value was dependent only on the total depth *n*.

When compared with the mutations already identified by Sanger sequencing, this first filtering was effective to detect those mutations **(Fig. S1)**.

After the first filtering, the selected SNVs were then classified into the targeted regions (coding region, non-coding region, splicing junction) and types of changes (nonsense mutation, missense mutation, insertion or deletion) **(** **Fig. 1** **)**. SNVs were then filtered against the sequences observed at over 1% in control subjects because most common *GJB2* deafness causing mutations so far found in Japanese had shown <1% allele frequencies in the control population **(Fig. S2)**. Then, the minimum cut off value for the depth was decided to be 230 for each 12-patient pool, based on the data obtained for all exons of the *GJB2, CDH23,* and *KCNQ4* genes by Sanger sequencing and parallel sequencing **(Fig. S2)**. For splice-site mutations, 24 possible candidates for causative mutations were selected because SNVs within +/−2base from the exon-intron junction site were considered to be important for splicing [21], [22]. After the application of all these filters, the candidate deafness causing mutations were selected, and verified by the subsequent Sanger sequencing. For missense mutations, the Polyphen2 [23] software program was applied to predict the influence on the protein structure by amino acid substitution. Family member genotypes were also used to validate the co-segregations of the deafness trait and the candidate mutations in individual families.

### Comparison with Another Algorithm for Pooled DNA Samples

We also analyzed all the data with VIPR, a program established and validated for use with pooled samples [24].

## Results

**Figure 2:**
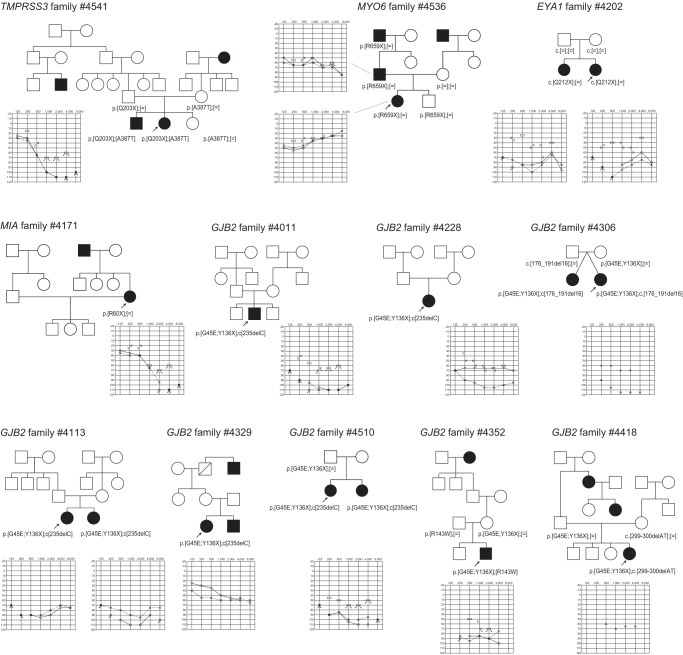
The pedigrees and audiograms of the patients with nonsense mutations after confirmation by Sanger sequencing.

Of 7 selected nonsense mutations, after Sanger sequencing, 2 were not confirmed but 5 actual nonsense mutations in 12 families were identified in *GJB2, EYA1, MIA, TMPRSS3,* and *MYO6* **(Table S2,** **Fig. 2** **)**.

**Figure 3:**
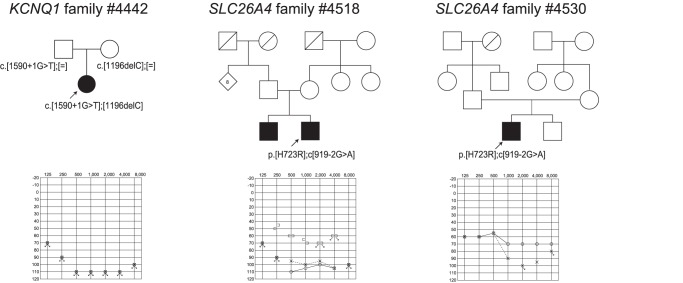
The pedigrees and audiograms of the patients with splice-site mutations after confirmation by Sanger sequencing.

Of 24 selected splice-site mutations, after Sanger sequencing, 22 were not identified but 2 actual splice-site mutations in 3 families were successfully identified in *KCNQ1* and *SLC26A4* **(Table S2,** **Fig. 3** **)**. The pathogenic nature was confirmed by 1) segregation within the family and 2) phenotypic configuration (long-QT for *KCNQ1* and enlarged vestibular aqueduct for *SLC26A4*).

**Figure 4:**
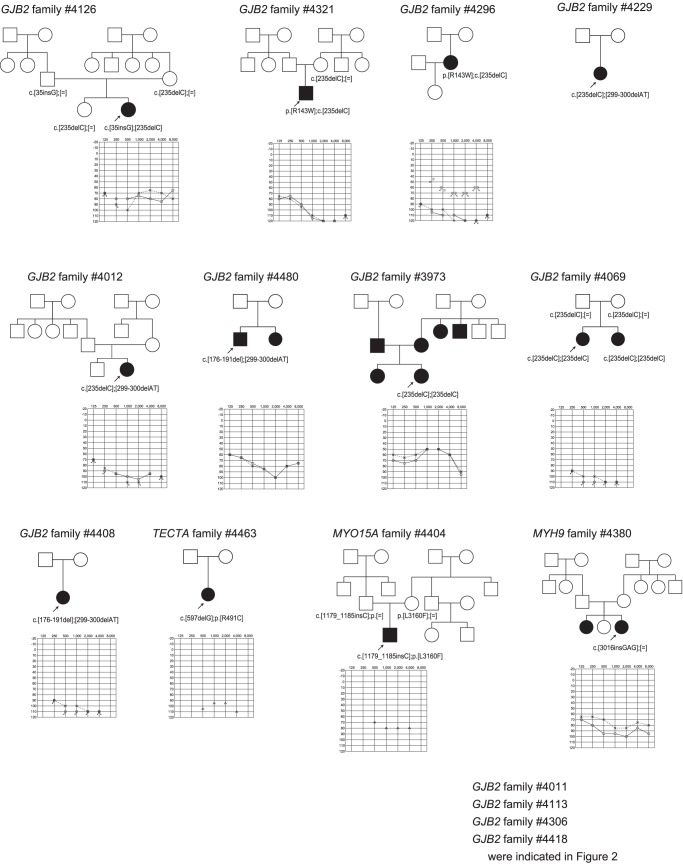
The pedigrees and audiograms of the patients with insertion-deletion mutations after confirmation by Sanger sequencing.

Of 27 selected insertion-deletion mutations, after Sanger sequencing, 6 actual mutations in 48 families were successfully identified in *GJB2, MYO15A* and *MYH9* **(Table S2,** **Fig. 4** **)**.

**Figure 5:**
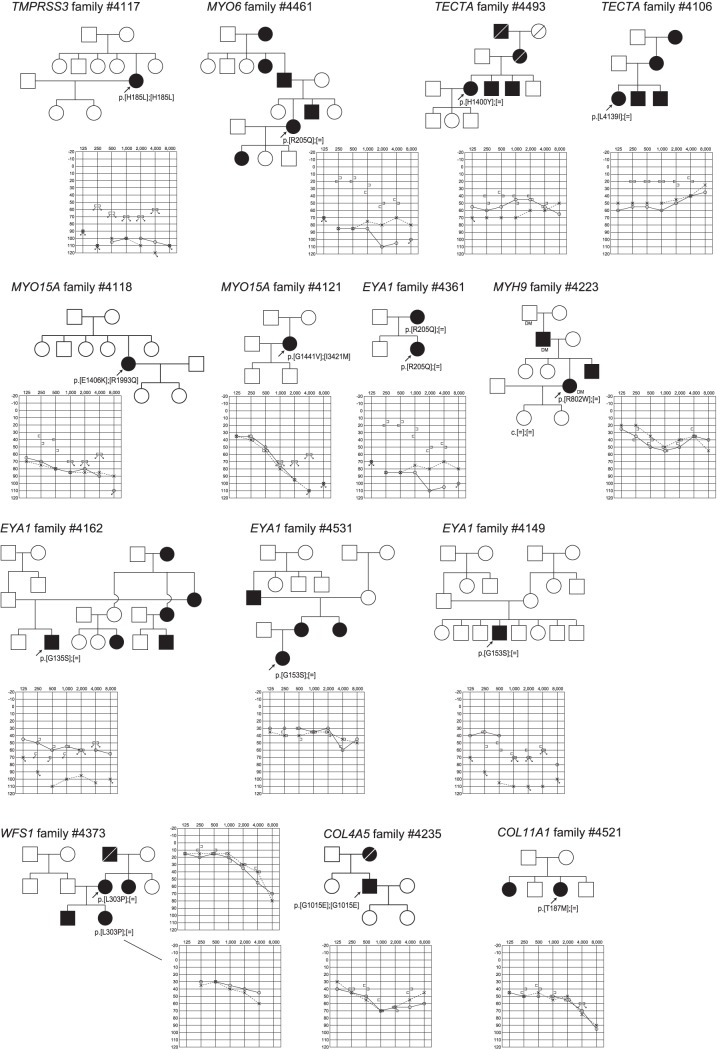
Examples of the families and audiograms of the patients with missense mutations after confirmation by Sanger sequencing.

Of 622 missense mutations, 254 mutations were confirmed by Sanger sequencing. By using PolyPhen2 software, 167 were classified as “damaging” or “probably damaging” or “possibly damaging” and 87 were categorized as “benign” (**Table S2**). Of 167 selected missense mutations 163 were <1% allele frequencies in both the 1000 genome project (http://www.1000genomes.org/node/home) and the NHLB grand opportunity exome sequencing project: 6500 exomes (http://esp.gs.washington.edu/drupal/). *TMPRSS3*, *MYO15A, GJB2, SLC26A4* were found to be responsible for deafness in autosomal recessive or sporadic families. Examples of the families are shown in **Fig. 5** **,** **6**. *TECTA, WFS-1, MYH9, EYA1, COL4A5, COL11A1* were identified as the responsible genes for deafness in autosomal dominant families (**Fig. 5** **,** **6**).

**Figure 6:**
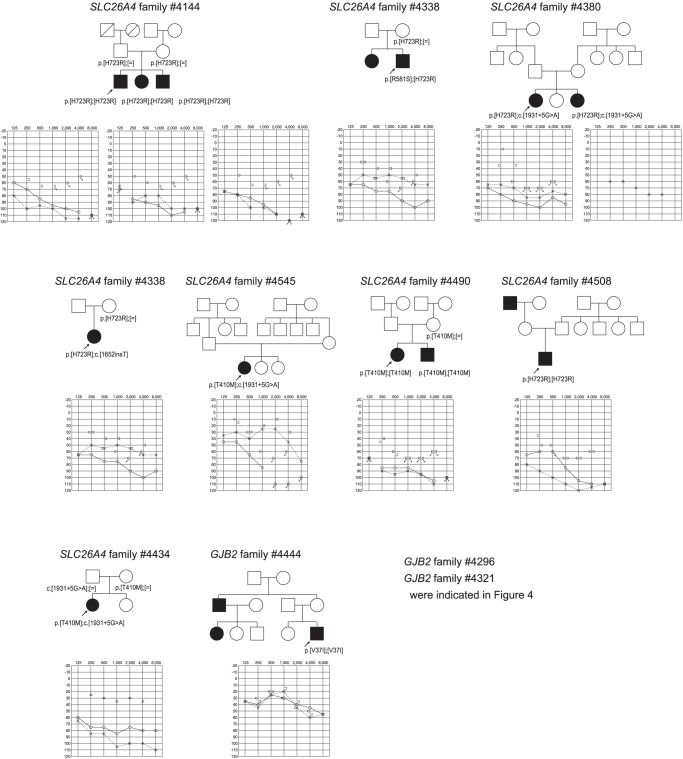
Examples of the families and audiograms of the patients with missense mutations after confirmation by Sanger sequencing.

**Figure 7:**
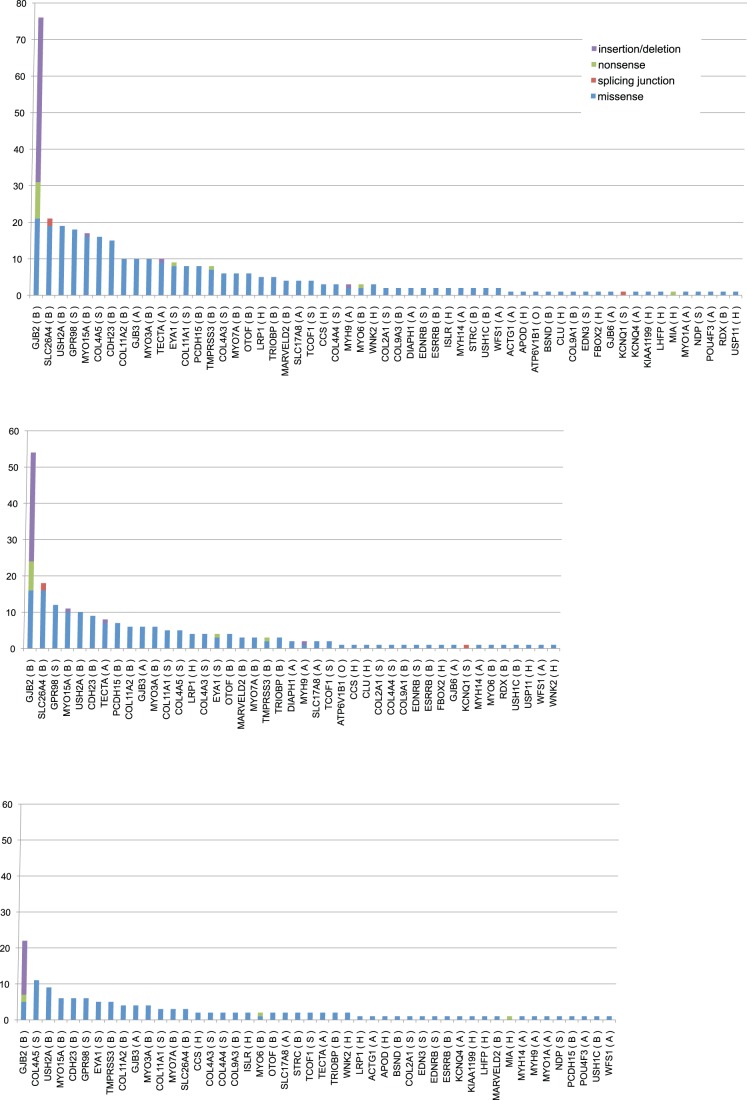
A: The number of mutations/mutation candidates indicating that the majority of the responsible gene mutations are accumulated in particular major causative genes. B: The number of mutations/mutation candidates in the early-onset group. C: The number of mutations/mutation candidates in the late-detected group.

As in **Table S2**, a total of 57 responsible genes were found, and the number of mutations/mutation candidates is shown in **Fig. 1**. *GJB2* was exceptionally higher, followed by *SLC26A4, USH2A, GPR98, MYO15A, COL4A5,* and *CDH23*. In the early-onset group, *GJB2*, *SLC26A4, GPR98, MYO15A, USH2A, CDH23, and TECTA* were frequently found, in contrast to the late-detected group, where *GJB2*, *COL4A5, USH2A, MYO15A, CDH23, GPR98, EYA1, and TMPRSS3* were frequently found (**Fig. 7**). The number of possible mutations in the early-onset group vs. late-detected group was 54∶22 for *GJB2*, 7∶1 for *PCDH15*, 8∶3 for *SLC26A4*, 18∶2 for *TECTA*, and 3∶5 for *TMPRSS3*.

Comparison data between the current algorithm and VIPR, which is widely used for pooled sample analysis due to its higher specificity in mutation detection compared to other programs for pooled samples, is shown in **Table S3.** VIPR is unable to detect deletion/insertion mutations, as well as some missense mutations. 93.5% (87/93) and 84.1% (37/44) of the mutations were detected in the *GJB2* and *SLC26A4* genes that had already been fully sequenced by Sanger sequencing, respectively.

## Discussion

With regard to sensitivity and specificity, we placed priority on sensitivity because one of the main purposes was clarifying genetic epidemiology. In addition, we used pooled DNA samples because a large number of sample is needed for genetic epidemiology. With the cut off value setting in this study, we could obtain high sensitivity (93.5% sensitivity on the basis of *GJB2, SLC26A4*)**(Fig. S1)**. We also analyzed all the data with VIPR, a program established and validated for use with pooled samples [24]. However, sensitivity (84.1%) was not as satisfactory as the current algorithm (**Table S3**). Also, because VIPR is unable to detect deletion/insertion mutations, we used our own algorithm in this study.

On the other hand, it is also true that one problem of the present algorithm is low specificity (high false positive rates: 16% for nonsense, 90% for splice-site, 75% for insertion-deletion mutations and 67% for missense mutations) necessitating time-consuming direct sequencing confirmation afterwards and making it unsuitable for diagnostic purposes. The low specificity was improved by using a more stringent cut off line in the minimum depth of coverage as well as a more stringent *p*-value in the binomial distribution filtering process. But for diagnosis, more sophisticated methods and algorithms with higher specificity such as bar-code procedures are available for genetic testing for individual patients.

With regard to five nonsense mutations in 12 families (identified in *GJB2, EYA1, MIA, TMPRSS3, MYO6*), two selected splice-site mutations in three families (identified in *KCNQ1* and *SLC26A4)*, and six insertion-deletion mutations (identified in *GJB2, MYO15A* and *MYH9)*, segregation analysis confirmed they are plausible disease causing mutations **(** **Fig. 2** **–** **4** **)**. For 163 selected missense mutations identified in 33 out of 54 known non-syndromic genes, it is difficult to reach a final conclusion about whether they are really disease causing mutations or not. Although some of the families were too small for segregation study or we failed to collect enough samples from familial members, most cases are consistent with the assumption that these are pathogenic mutations based on the software programs to predict the influence on the protein structure [20]. Actual causative mutations were successfully identified from the selected recessive as well as dominant families in which all the samples of family members were collected (Examples are shown in **Fig. 5** **,** **6**). *TMPRSS3*, *MYO15A, GJB2, SLC26A4* were found to be responsible for deafness in autosomal recessive or sporadic families, while *TECTA, WFS1, MYH9, EYA1, COL4A5* and *COL11A1* were identified as the responsible genes for deafness in autosomal dominant families.

One interesting result is that a mutation in a novel putative responsible deafness gene, *MIA*, which is highly expressed in the inner ear, was identified in a dominant family (#4171), in the present study. Although the detailed function in the inner ear is currently unknown, genes that are highly expressed in the inner ear, as revealed by cDNA microarray analysis, may have a crucial functional role there [18].

The other interesting result was the mutations in the genes previously reported to be syndromic genes such as *EYA1*. Although re-contact was not possible in all cases, detailed genotype/phenotype correlation study will be an open question. One family was later found to be associated with ear pits (diagnosed as BOR syndrome) (family #4361 in Fig. 5), but the rest of the contacted families did not have any associated branchial disclosure. Interestingly, all families were associated with inner ear anomaly, and therefore these families have slightly different clinical phenotype from typical BOR syndrome. As in this case, the mutation analysis using MPS will potentially expand the phenotypic variations.

Based on the sensitivity, nonsense mutations, splice-site mutations, insertion-deletion mutations or selected missense mutations were found in 57 out of 112 genes (33/56 non-syndromic genes, 12/22 syndromic genes, and 12/36 genes highly expressed in the cochlea). The mutations previously found in Invader assays or direct sequencing were also confirmed effectively in our MPS algorithm. Of 93 previously found *GJB2* and *SLC26A4* mutations, we confirmed 87 (93.5%) of them (Table S3). Approximately 86.6% (187/216) of the patients had at least one mutation.

Of the 187 patients, in 69 the etiology of the hearing loss was completely explained (biallelic probably pathogenic mutations in autosomal recessive or sporadic cases, or one probably pathogenic mutation in autosomal dominant cases), and in 12 was possibly explained (two mutations with one probably pathogenic mutation and an unknown variant in the same gene in autosomal recessive or sporadic cases, or one unknown mutation in autosomal dominant cases).

A noteworthy result obtained in this study was that the data clarified the molecular epidemiology for deafness in our population. For two decades, there have been extensive efforts to identify the etiology of deafness and those studies have determined that genetic causes are commonly involved in congenital/early-onset sensorineural hearing loss, but there has been no etiological data on a genetic basis using a large number of patients. It has been reported that more than 100 loci and 46 causative genes are causing deafness [25]. To evaluate which genes have an impact on deafness epidemiology, the number of mutations/mutation candidates was counted. Among the identified mutations, the number of *GJB2* mutations was exceptionally higher at 80 alleles, followed by those in *SLC26A4, USH2A, GPR98, MYO15A, COL4A5,* and *CDH23* (**Fig. 7**). Regarding the number of possible mutations in each gene, *GJB2* (54∶22), *PCDH15* (7∶1), *SLC26A4* (18∶3), *TECTA* (8∶2) were frequent in the early-onset group. In contrast, *TMPRSS3* (3∶5) was predominantly found in the late-detected (based on the age of awareness) group. Such tendency is in line with reported phenotypes.

Actually, detected mutations were confirmed to be pathogenic in selected families (**Fig. 2** **–** **6**). Although *USH2A* and *GPR98* (which underly Usher syndrome type 2) mutations were great in number, this is to be expected based on the extremely large size of the gene.

An important fact is that the samples we used were collected randomly from 33 different hospitals distributed throughout Japan, therefore we believe them to be a representative cohort of Japanese patients and suitable for epidemiological evaluation. We have developed an advanced screening strategy focusing on frequently recurring mutations that are most likely to be encountered in the clinical setting that identifies approximately 40% of deafness patients [5]. This indicates that 30–40% of patients have deafness due to recurrent mutations in particular genes, such as *GJB2* or *SLC26A4*. In fact, 25% (53/216 overall), and 42% (50/120 for early-onset) of the patients were diagnosed by those recurrent mutations. *GJB2* has been known as the most prevalent responsible gene for deafness worldwide and 14–16% (25–26% for congenital cases) of Japanese hearing loss patients have *GJB2* mutations [5], [26]. Mutations in *SLC26A4, MYO15A,* and *CDH23* are also reported to be frequent and important causes of deafness [5], [25]. The number of mutations of *GJB2* is actually the highest among the genes in the mutation database (**Fig. 7**), supporting the view that the majority of the responsible gene mutations are such commonly found ones with the remainder being various rare genes/mutations. Those genes have not usually been screened and therefore mutations in them have not been diagnosed by the conventional approach. From that point of view, MPS has the potential to identify such rare genes/mutations.

In conclusion, MPS enabled us to discover rare causative genes for a highly heterogeneous monogenic disease and revealed the genetic epidemiology of deafness. This epidemiologic data will shed light on gene evolution and provide the basis for future genetic screening strategies.

## Supporting Information

Figure S1 **The validity of the binomial distribution filter used in this study.** The horizontal axis indicates depth of coverage of each SNV detected by MPS analysis and the vertical axis indicates calculated allele frequency in each 12-patient pool (calculated by alternative base read number divided by total (alternative+reference) base read number for each SNV). Mutations of the known three genes, *GJB2, KCNQ4,* and *CDH23* either by MPS (circle) or Sanger sequencing (dot). Red: *CDH23,* Blue: *GJB2,* Green: *KCNQ4.* The cut-off line using first filtering algorithm is indicated by a black line. Most of the SNVs detected by Sanger sequencing were distributed above the threshold indicating that mutations selected are effectively identified. *GJB2* (Blue) had a deeper depth which means MPS data is more reliable whereas *KCNQ4* (Green) had shallow depth, which is less reliable. Actually Sanger sequencing (dot) showed reasonable data.
(PDF)

Figure S2 **A:** The ROC curve for the optimal cut-off value of the allele frequency at each nucleotide position using the data obtained for all exons of the *GJB2, CDH23,* and *KCNQ4* genes by Sanger sequencing. **B:** The ROC curve for the optimal cut-off value of the depth at each nucleotide position using the data obtained for all exons of the *GJB2, CDH23,* and *KCNQ4* genes by Sanger sequencing.
(PDF)

Table S1 **One hundred twelve potentially deafness-causative genes, including 54 reported causative non-syndromic hearing loss genes, 22 reported causative syndromic hearing loss genes, and 36 genes that are highly expressed in the inner ear.**
(PDF)

Table S2 **Mutations/mutation candidates confirmed by Sanger sequencing.** Nonsense mutations, splice-site mutations, or missense mutations were found in 57 out of 112 genes.
(PDF)

Table S3 **Comparison of data between the current algorithm and VIPR.** 93.5% (87/93) and 84.1% (37/44) of the mutations was detected in *GJB2* and *SLC26A4* genes already fully sequenced by Sanger sequencing, respectively.
(PDF)
