# Supplementary figures and images for: Targeted Exon Sequencing Successfully Discovers Rare Causative Genes and Clarifies the Molecular Epidemiology of Japanese Deafness Patients
Source: PLoS One. 2013 Aug 13;8(8):e71381. doi: 10.1371/journal.pone.0071381 (PMC3742761; doi:10.1371/journal.pone.0071381)

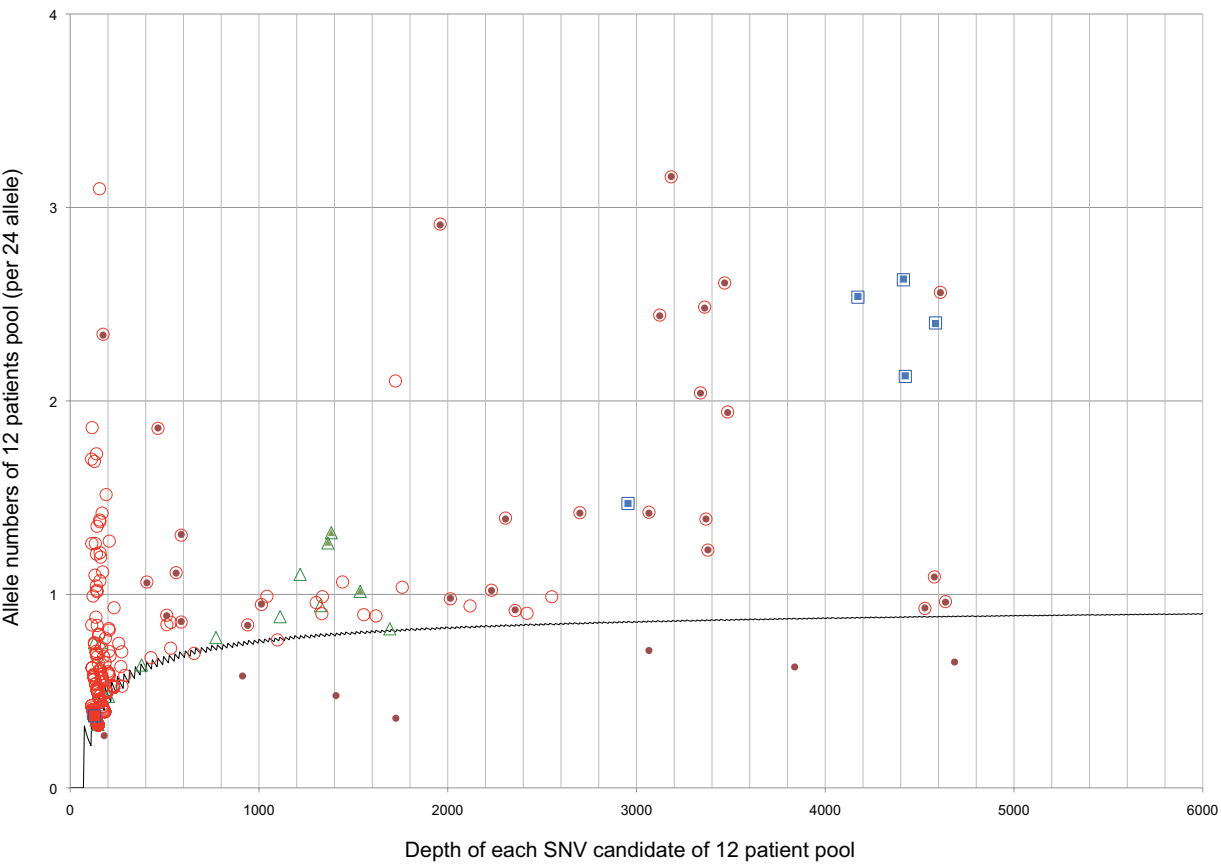

Supplement: Figure S1 — The validity of the binomial distribution filter used in this study. The horizontal axis indicates depth of coverage of each SNV detected by MPS analysis and the vertical axis indicates calculated allele frequency in each 12-patient pool (calculated by alternative base read number divided by total (alternative+reference) base read number for each SNV). Mutations of the known three genes, GJB2, KCNQ4, and CDH23 either by MPS (circle) or Sanger sequencing (dot). Red: CDH23, Blue: GJB2, Green: KCNQ4. The cut-off line using first filtering algorithm is indicated by a black line. Most of the SNVs detected by Sanger sequencing were distributed above the threshold indicating that mutations selected are effectively identified. GJB2 (Blue) had a deeper depth which means MPS data is more reliable whereas KCNQ4 (Green) had shallow depth, which is less reliable. Actually Sanger sequencing (dot) showed reasonable data. (PDF) [file pone.0071381.s001.pdf]

A

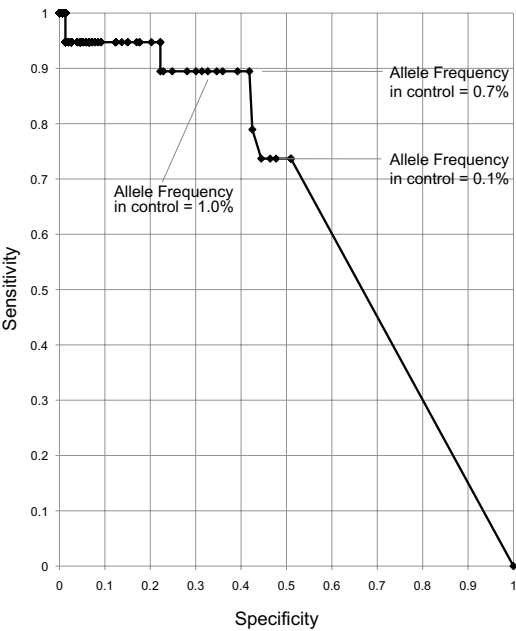

B

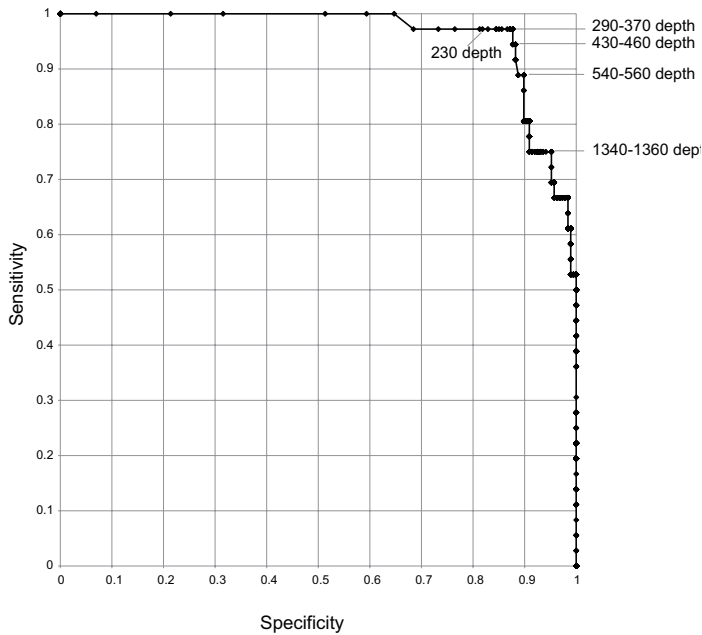

Supplement: Figure S2 — A: The ROC curve for the optimal cut-off value of the allele frequency at each nucleotide position using the data obtained for all exons of the GJB2, CDH23, and KCNQ4 genes by Sanger sequencing. B: The ROC curve for the optimal cut-off value of the depth at each nucleotide position using the data obtained for all exons of the GJB2, CDH23, and KCNQ4 genes by Sanger sequencing. (PDF) [file pone.0071381.s002.pdf]
