# Supplementary material for: Targeted Exon Sequencing Successfully Discovers Rare Causative Genes and Clarifies the Molecular Epidemiology of Japanese Deafness Patients
Source: PLoS One. 2013 Aug 13;8(8):e71381. doi: 10.1371/journal.pone.0071381 (PMC3742761; doi:10.1371/journal.pone.0071381)
Supplement: Table S1 — One hundred twelve potentially deafness-causative genes, including 54 reported causative non-syndromic hearing loss genes, 22 reported causative syndromic hearing loss genes, and 36 genes that are highly expressed in the inner ear. (PDF) [file pone.0071381.s003.pdf]

Supplementary Table S1 . One hundred twelve potentially deafness-causative genes.

Categoly	Locus Symbol	Gene Symbol	Gene ID	Transcript Variant
**nonsyndromic deafness**	DFNA1	DIAPH1	NM_005219.4	NM_001079812.2
	DFNA2	KCNQ4	NM_004700.2	NM_172163.2
	DFNA2	GJB3	NM_024009.2	NM_001005752.1
	DFNA3	GJB6	NM_006783.4	NM_001110219.2
				NM_001110220.2
				NM_001110221.2
	DFNA4	MYH14	NM_024729.3	NM_001145809.1
				NM_001077186.1
	DFNA5	DFNA5	NM_004403.2	NM_001127454.1
				NM_001127453.1
	DFNA6/14/38	WFS1	NM_006005.3	NM_001145853.1
	DFNA8/12/DFNB21	TECTA	NM_005422.2	-
	DFNA9/31	COCH	NM_004086.2	NM_001135058.1
	DFNA10	EYA4	NM_004100.4	NM_172103.3
				NM_172105.3
	DFNA11/DFNB2/USH1B	MYO7A	NM_000260.3	NM_001127179.2
				NM_001127180.1
	DFNA13/DFNB53/STL3	COL11A2	NM_080680.2	NM_080679.2
				NM_080681.2
				NM_001163771.1
	DFNA15	POU4F3	NM_002700.1	-
	DFNA17	MYH9	NM_002473.3	-
	DFNA20/DFNA26	ACTG1	NM_001614.2	NM_001199954.1
	DFNA22/DFNB37	MYO6	NM_004999.3	-
	DFNA23/BOR3	SIX1	NM_005982.3	-
	DFNA25	SLC17A8	NM_139319.2	NM_001145288.1
	DFNA28	GRHL2	NM_024915.3	-
	DFNA36/DFNB7/11	TMC1	NM_138691.2	-
	DFNA39	DSPP	NM_014208.3	-
	DFNA40	CRYM	NM_001888.2	NM_001014444.2

DFNA44	CCDC50	NM_178335.2	NM_174908.3
DFNA48	MYO1A	NM_005379.2	-
DFNA50	MIR96	NR_029512.1	
DFNB1/DFNA3	GJB2	NM_004004.5	-
DFNB3	MYO15A	NM_016239.3	-
DFNB4/Pendred	SLC26A4	NM_000441.1	-
DFNB6	TMIE	NM_147196.2	-
DFNB8/10	TMPRSS3	NM_024022.1	NM_032405.1
DFNB9	OTOF	NM_194248.2	NM_194322.2
			NM_194323.2
			NM_004802.3
DFNB12/USH1D	CDH23	NM_022124.4	NM_001171930.1
			NM_001171931.1
			NM_001171932.1
			NM_001171933.1
			NM_001171934.1
			NM_001171936.1
			NM_052836.3
DFNB16	STRC	NM_153700.2	-
DFNB18/USH1C	USH1C	NM_153676.2	NM_005709.3
DFNB22	OTOA	NM_144672.3	NM_001161683.1
			NM_170664.2
DFNB23/USH1F	PCDH15	NM_033056.3	NM_001142763.1
			NM_001142764.1
			NM_001142765.1
			NM_001142766.1
			NM_001142767.1
			NM_001142769.1
			NM_001142770.1
			NM_001142771.1
			NM_001142772.1
			NM_001142773.1

DFNB24	RDX	NM_002906.3	-
DFNB25	GRXCR1	NM_001080476.1	-
DFNB28	TRIOBP	NM_007032.5	NM_138632.2
			NM_001039141.2
DFNB29	CLDN14	NM_144492.1	NM_001146077.1
			NM_001146078.1
			NM_001146079.1
			NM_012130.2
DFNB30	MYO3A	NM_017433.4	-
DFNB31/USH2D	WHRN	NM_015404.2	
DFNB35	ESRRB	NM_004452.2	-
DFNB36	ESPN	NM_031475.2	-
DFNB39	HGF	NM_000601.4	NM_001010931.1
			NM_001010932.1
			NM_001010933.1
			NM_001010934.1
DFNB49	MARVELD2	NM_001038603.2	NM_001244734.1
DFNB59	PJVK	NM_001042702.3	
DFNB61	SLC26A5	NM_206883.1	NM_001167962.1
			NM_206884.2
			NM_206885.2
			NM_198999.2
DFNB63	LRTOMT	NM_001145307.1	NM_001145308.2
			NM_001205138.1
			NM_145309.3
DFNB66/67	LHFPL5	NM_182548.3	-
DFNB73	BSND	NM_057176.2	-
DFNB77	LOXHD1	NM_144612.5	NM_001173129.1
			NM_001145472.2
			NM_001145473.2
DFNB79	TPRN	NM_001128228.2	-
DFNB84	PTPRQ	NM_001145026.1	-

**Alport**		COL4A5	NM_000495.3	NM_033380.1
		COL4A3	NM_000091.4	-
		COL4A4	NM_000092.4	-
**BOR**	BOR1	EYA1	NM_000503.4	NM_172058.2
				NM_172059.2
				NM_172060.2
	BOR2	SIX5	NM_175875.4	-
**Jervell & Lange-Nielsen Syndrome**	JLNS1	KCNQ1	NM_000218.2	-
	JLNS2	KCNE1	NM_000219.3	NM_001127668.1
				NM_001127669.1
				NM_001127670.1
**Norrie Disease**	NDP	NDP	NM_000266.3	-
**Pendred Syndrome**	PDS	FOXI1	NM_012188.4	NM_144769.2
**Stickler Syndrome**	STL1	COL2A1	NM_001844.4	NM_033150.2
	STL2	COL11A1	NM_001854.3	NM_001190709.1
				NM_080629.2
				NM_080630.3
**Treacher Collins Syndrome**	TCOF1	TCOF1	NM_000356.3	NM_001195141.1
				NM_001135243.1
				NM_001135244.1
				NM_001135245.1
				NM_001008657.2
**Usher Syndrome**	USH1G	USH1G	NM_173477.2	-
	USH2A	USH2A	NM_206933.2	NM_007123.5
	USH2C	GPR98	NM_032119.3	-
	USH3	CLRN1	NM_174878.2	NM_052995.2
				NM_001195794.1
**Waardenburg Syndrome**	WS1/WS3	PAX3	NM_181457.3	NM_001127366.2
				NM_181458.3
				NM_181459.3
				NM_181460.3

				NM_181461.3
	WS2A	MITF	NM_000248.3	NM_001184967.1
				NM_001184968.1
				NM_006722.2
				NM_198158.2
				NM_198159.2
				NM_198177.2
				NM_198178.2
	WS2D	SNAI2	NM_003068.4	-
	WS4	EDNRB	NM_000115.3	NM_003991.3
				NM_001122659
				NM_001201397.1
	WS4	EDN3	NM_000114.2	NM_207032.1
				NM_207033.1
				NM_207034.1
	WS4	SOX10	NM_006941.3	-
**Highly Expression genes**		COL9A1	NM_001851.4	NM_078485.3
		PTGDS	NM_000954.5	-
		OTOR	NM_020157.2	-
		ZP2	NM_003460.1	-
		CLU	NM_203339.2	NR_038335.1
		APOD	NM_001647.3	-
		KIAA1199	NM_018689.1	-
		FBXO2	NM_012168.4	-
		RBP1	NM_002899.3	NM_001130992.1
				NM_001130993.1
		ATP1A2	NM_000702.3	-
		LHFP	NM_005780.3	-
		UBA52	NM_003333.3	NM_001033930.1
		IGFBP6	NM_002178.2	-

S100A13	NM_005979.2	NM_001024210.1
		NM_001024211.1
		NM_001024212.1
		NM_001024213.1
FOXD1	NM_004472.2	-
MGP	NM_000900.2	NM_001190839.1
MIA	NM_006533.2	NM_001202553.1
COL9A3	NM_001853.3	-
DGCR6	NM_005675.4	-
PCDHGC3	NM_002588.2	NM_032402.1
		NM_032403.1
VAMP5	NM_006634.2	-
WNK2	NM_006648.3	-
CA14	NM_012113.1	-
CCS	NM_005125.1	-
PLEKHB1	NM_021200.2	NM_001130033.1
		NM_001130034.1
		NM_001130035.1
		NM_001130036.1
C19orf53	NM_014047.2	-
SELM	NM_080430.2	-
MT3	NM_005954.2	-
HYI	NM_031207.2	NM_001190880.2
		NM_001243526.1
ISLR	NM_005545.3	NM_201526.1
DDR2	NM_006182.2	NM_001014796.1
UTY	NM_007125.3	NM_182659.1
		NM_182660.1
USP11	NM_004651.3	-
ZNF288	NM_015642.3	
ATP6V1B1	NM_001692.3	-
LRP1	NM_002332.2	-
