# Supplementary material for: Targeted Exon Sequencing Successfully Discovers Rare Causative Genes and Clarifies the Molecular Epidemiology of Japanese Deafness Patients
Source: PLoS One. 2013 Aug 13;8(8):e71381. doi: 10.1371/journal.pone.0071381 (PMC3742761; doi:10.1371/journal.pone.0071381)
Supplement: Table S2 — Mutations/mutation candidates confirmed by Sanger sequencing. Nonsense mutations, splice-site mutations, or missense mutations were found in 57 out of 112 genes. (PDF) [file pone.0071381.s004.pdf]

Supplementary Table 2. Identified causative mutations and/or mutation candidates.

Nonsense mutations

No.	gene symbol	mRNA accession number	protein accession number	base change	aa change	Average depth	Result of next generation sequencing: Early onset HL patients (in 240 alleles)	Result of next generation sequencing: Late onset HL patients (in 192 alleles)	Result of next generation sequencing: control (in 144 alleles)	Result of direct sequencing (confirmed allele number in 432 patient alleles)	Hereditary form of the mutation detected families	Accession number
1	EYA1	NM_000503.3	NP_000494.2	c.634C>T	p.Q212X	2515	1	0	0	1	AR	AB838988
2	GJB2	NM_004004.4	NP_003995.2	c.408C>A	p.Y136X	4110	8.5	2.6	0	10	AR, sporadic	AB838989
3	MIA	NM_006533.2	NP_006524.1	c.178C>T	p.R60X	1826	0	1.1	0	1	AD	AB838990
4	MYO6	NM_004999.3	NP_004990.3	c.1975C>T	p.R659X	923	0	0.9	0	1	AD	AB838991
5	TMPRSS3	NM_024022.1	NP_076927.1	c.607C>A	p.Q203X	1207	1.1	0	0	1	AR	AB838992

Splicing junction mutations

No.	gene symbol	mRNA accession number	protein accession number	base change	aa change	Average depth	Result of next generation sequencing: Early onset HL patients (in 240 alleles)	Result of next generation sequencing: Late onset HL patients (in 192 alleles)	Result of next generation sequencing: control (in 144 alleles)	Result of direct sequencing (confirmed allele number in 432 patient alleles)	Hereditary form of the mutation detected families	Accession number
1	KCNQ1	NM_000218.2	NP_000209.2	c.1590+1G>T	p.Q530fs	1348	0.9	0	0	1	sporadic	AB838993
2	SLC26A4	NM_000441.1	NP_000432.1	c.919-2A>G	p.V306fs	3891	2.1	0	0	2	AR, sporadic	AB838994

Insertion/Deletion mutations

No.	gene symbol	mRNA accession number	protein accession number	base change	aa change	Average depth	Result of next generation sequencing: Early onset HL patients (in 240 alleles)	Result of next generation sequencing: Late onset HL patients (in 192 alleles)	Result of next generation sequencing: control (in 144 alleles)	Result of direct sequencing (confirmed allele number in 432 patient alleles)	Hereditary form of the mutation detected families	Accession number
1	GJB2	NM_004004.4	NP_003995.2	c.36insG	p.V12fs	404	3.7	0	0	1	sporadic	AB838995
2	GJB2	NM_004004.4	NP_003995.2	c.176-191del16	p.G59fs	299	13.5	0	0	3	AR, sporadic	AB838996
3	GJB2	NM_004004.4	NP_003995.2	c.235delC	p.L79fs	740	18.8	14.4	8.8	36	AR, sporadic, unknown	AB838997
4	GJB2	NM_004004.4	NP_003995.2	c.299-300delAT	p.H100fs	407	32.1	0	0	5	AR, sporadic	AB838998
5	MYO15A	NM_016239.3	NP_057323.3	c.1179-1185insC	p.P393fs	493	3.2	0	0	1	sporadic	AB838999
6	MYH9	NM_002473.3	NP_002464.1	c.3016insGAG	p.T1006fs	230	3.8	0	0	1	AR	AB839000
7	TECTA	NM_005422.2	NP_005413.2	c.597delG	p.L199fs	507	1.8	0	0	1	sporadic	AB839001

Missense mutations

No.	gene symbol	mRNA accession number	protein accession number	base change	aa change	Average depth	Result of next generation sequencing: Early onset HL patients (in 240 alleles)	Late onset HL patients (in 192 alleles)	control (in 144 alleles)	Result of direct sequencing (confirmed allele number in 432 patient alleles)	Polyphen2 score	Hereditary form of the mutation detected families	Accession number
1	ACTG1	NM_001614.2	NP_001605.1	c.485C>T	p.T162M	853	0	1	0	1	0.995	sporadic	AB839002
2	APOD	NM_001647.2	NP_001638.1	c.91C>T	p.P31S	1371	0	1	0	1	0.992	AD	AB839003
3	ATP6V1B1	NM_001692.3	NP_001683.2	c.599T>C	p.I200T	607	1.2	0	0	1	1	unknown	AB839004
4	BSND	NM_057176.2	NP_476517.1	c.893G>A	p.G298E	1924	0	1.4	0	1	1	sporadic	AB839005
5	CCS	NM_005125.1	NP_005116.1	c.167C>T	p.T56I	1331	0	0.8	0	1	0.919	sporadic	AB839006
6	CCS	NM_005125.1	NP_005116.1	c.212G>A	p.R71Q	1140	0	1.2	0	1	1	sporadic	AB839007
7	CCS	NM_005125.1	NP_005116.1	c.586C>T	p.R196C	776	1.2	0	0	1	1	sporadic	AB839008
8	CDH23	NM_022124.3	NP_071407.3	c.719C>T	p.P240L	4102	1.4	1.3	1	2	0.825	AD/Mit, AR, sporadic	AB839009
9	CDH23	NM_022124.3	NP_071407.3	c.2866G>A	p.E956K	511	0.9	0	0	1	1	sporadic	AB839010
10	CDH23	NM_022124.3	NP_071407.3	c.4249C>T	p.R1417W	1293	0	1.1	0	1	0.986	unknown	AB839011
11	CDH23	NM_022124.3	NP_071407.3	c.4346G>A	p.G1449D	1090	0	1.1	0	1	1	sporadic	AB839012
12	CDH23	NM_022124.3	NP_071407.3	c.4762C>T	p.R1588W	525	5.1	4.5	0	8	1	AR, AD, sporadic	AB839013
13	CDH23	NM_022124.3	NP_071407.3	c.7145G>A	p.R2382Q	2357	0.9	0	0	1	0.972	sporadic	AB839014
14	CDH23	NM_022124.3	NP_071407.3	c.8734G>A	p.G2912S	2014	1	0	0	1	0.999	sporadic	AB839015
15	CLU	NM_001831.2	NP_001822.2	c.382G>A	p.V128I	2367	1.1	0	0	1	0.973	AR	AB839016
16	COL11A1	NM_080629.2	NP_542196.2	c.560C>T	p.T187M	4518	1.2	0	0	3	0.998	AD/Mit	AB839017
17	COL11A1	NM_080629.2	NP_542196.2	c.2614T>A	p.F872I	1533	1.9	1.5	0	4	0.996	AD, sporadic	AB839018
18	COL11A1	NM_080629.2	NP_542196.2	c.2771C>T	p.P924L	2254	1	3.1	0	1	0.845	sporadic	AB839019
19	COL11A2	NM_080680.2	NP_542411.2	c.688G>T	p.G230W	3158	3.4	3.2	0	7	0.987	AD/Mit, sporadic	AB839020
20	COL11A2	NM_080680.2	NP_542411.2	c.2002C>T	p.P668S	1072	1.4	0	0	1	0.991	AD/Mit, AR, sporadic	AB839021
21	COL11A2	NM_080680.2	NP_542411.2	c.3392G>A	p.R1131Q	1146	1	0	0	1	0.996	AD	AB839022
22	COL11A2	NM_080680.2	NP_542411.2	c.4265C>T	p.P1422L	541	0	1.1	1.2	1	0.852	sporadic	AB839023
23	COL2A1	NM_001844.4	NP_001835.3	c.4148C>T	p.T1383M	2858	1.8	0	0	1	0.761	unknown	AB839024
24	COL2A1	NM_001844.4	NP_001835.3	c.4196A>G	p.Y1399C	2465	0	1	0	1	0.928	sporadic	AB839025
25	COL4A3	NM_000091.3	NP_000082.2	c.469G>C	p.G157R	771	0.9	0	0	1	1	AD	AB839026
26	COL4A3	NM_000091.3	NP_000082.2	c.1295C>T	p.P432L	1491	1.6	2.5	0	3	1	AD, sporadic	AB839027
27	COL4A3	NM_000091.3	NP_000082.2	c.2827G>A	p.G943R	1148	0.8	0	0	1	1	sporadic	AB839028
28	COL4A3	NM_000091.3	NP_000082.2	c.4928G>A	p.R1643K	2512	1	0	0	1	0.794	AD	AB839029
29	COL4A4	NM_000092.4	NP_000083.3	c.2045A>G	p.D682G	1734	0	1.5	0	1	0.669	unknown	AB839030
30	COL4A4	NM_000092.4	NP_000083.3	c.2872C>G	p.P958A	2605	0	1.1	0	1	0.611	sporadic	AB839031
31	COL4A4	NM_000092.4	NP_000083.3	c.4316G>A	p.G1439D	848	3.9	0	0	1	0.993	AR	AB839032

32	COL4A5	NM_033380.1	NP_203699.1	c.2858G>T	p.G953V	1712	4.9	8.3	0	14	1	AD, AD/Mit, sporadic	AB839033
33	COL4A5	NM_033380.1	NP_203699.1	c.3044G>A	p.G1015E	877	0	0.8	0	1	1	AD/Mit	AB839034
34	COL4A5	NM_033380.1	NP_203699.1	c.4003C>T	p.P1335S	1706	0	1.5	0	1	0.873	sporadic	AB839035
35	COL9A1	NM_001851.3	NP_001842.3	c.2395G>C	p.G799R	738	1.1	0	0	1	1	sporadic	AB839036
36	COL9A3	NM_001853.3	NP_001844.3	c.1361G>A	p.G454E	1599	0	1.4	0	1	1	sporadic	AB839037
37	COL9A3	NM_001853.3	NP_001844.3	c.1649C>T	p.P550L	733	0	1.6	0.6	1	1	AD	AB839038
38	DIAPH1	NM_005219.3	NP_005210.3	c.2032C>T	p.P678S	826	1.3	0	0	2	0.987	AD/Mit	AB839039
39	EDN3	NM_000114.2	NP_000105.1	c.554C>T	p.T185M	930	0	0.8	0	1	1	sporadic	AB839040
40	EDNRB	NM_000115.1	NP_000106.1	c.311A>T	p.N104I	3279	0.9	1	0	2	0.997	sporadic	AB839041
41	ESRRB	NM_004452.2	NP_004443.2	c.475C>T	p.R159C	450	0.6	0	0	1	1	sporadic	AB839042
42	ESRRB	NM_004452.2	NP_004443.2	c.536G>A	p.R179H	1393	0	0.9	0	1	0.989	sporadic	AB839043
43	EYA1	NM_000503.3	NP_000494.2	c.403G>A	p.G135S	2170	0	2.3	0	4	1	AD/Mit, AD, sporadic	AB839044
44	EYA1	NM_000503.3	NP_000494.2	c.671G>T	p.G224V	3528	2.2	0.9	0	3	0.947	sporadic	AB839045
45	EYA1	NM_000503.3	NP_000494.2	c.1286A>G	p.D429G	3036	1.3	0	0	1	0.988	AD/Mit	AB839046
46	FBXO2	NM_012168.4	NP_036300.2	c.286G>A	p.G96R	242	0.7	0	0	1	1	AD/Mit	AB839047
47	GJB2	NM_004004.4	NP_003995.2	c.23C>T	p.T8M	2700	0	1.4	0	1	0.775	unknown	AB839048
48	GJB2	NM_004004.4	NP_003995.2	c.109G>A	p.V37I	3039	4.7	3.2	0	6	1	sporadic	AB839049
49	GJB2	NM_004004.4	NP_003995.2	c.134G>A	p.G45E	3213	8	2.4	1	10	1	AR, sporadic, AD/Mit/AR	AB839050
50	GJB2	NM_004004.4	NP_003995.2	c.257C>G	p.T86R	1960	2.9	0	0	1	1	unknown	AB839051
51	GJB2	NM_004004.4	NP_003995.2	c.427C>T	p.R143W	3822	2.5	0	0	3	1	sporadic	AB839052
52	GJB3	NM_001005752.1	NP_001005752.1	c.250G>A	p.V84I	2108	5	4	0	8	0.946	sporadic	AB839053
53	GJB3	NM_001005752.1	NP_001005752.1	c.437T>C	p.L146P	2029	2.4	0	0	1	1	unknown	AB839054
54	GJB3	NM_001005752.1	NP_001005752.1	c.459G>T	p.W153C	2618	0	1.1	0	1	0.999	sporadic	AB839055
55	GJB6	NM_006783.2	NP_006774.2	c.556A>G	p.T186A	5287	0.9	0	0	1	0.999	sporadic	AB839056
56	GPR98	NM_032119.3	NP_115495.3	c.913T>C	p.Y305H	4213	1.2	0	0	1	1	sporadic	AB839057
57	GPR98	NM_032119.3	NP_115495.3	c.1797A>T	p.R599S	2307	0.9	0	0	1	1	AR	AB839058
58	GPR98	NM_032119.3	NP_115495.3	c.1804G>A	p.A602T	2393	1.7	1.6	0.8	2	1	AR, sporadic	AB839059
59	GPR98	NM_032119.3	NP_115495.3	c.2039A>G	p.D680G	2847	1	2.1	0	2	1	sporadic	AB839060
60	GPR98	NM_032119.3	NP_115495.3	c.4703G>A	p.S1568N	2659	0	1.1	0	1	1	sporadic	AB839061
61	GPR98	NM_032119.3	NP_115495.3	c.6469G>A	p.V2157M	2717	1.4	0	0	1	0.872	sporadic	AB839062
62	GPR98	NM_032119.3	NP_115495.3	c.12335T>G	p.L4112W	1917	0	0.9	0	1	0.978	sporadic	AB839063
63	GPR98	NM_032119.3	NP_115495.3	c.12554G>C	p.G4185A	3516	0.9	0	0.8	1	0.999	sporadic	AB839064
64	GPR98	NM_032119.3	NP_115495.3	c.12704A>G	p.Y4235C	3269	1.6	0	0	2	0.994	sporadic	AB839065
65	GPR98	NM_032119.3	NP_115495.3	c.13763G>A	p.G4588E	3519	1.1	0	0	1	0.959	sporadic	AB839066
66	GPR98	NM_032119.3	NP_115495.3	c.13996A>G	p.I4666V	1437	0.9	0	0.9	1	0.615	AD	AB839067
67	GPR98	NM_032119.3	NP_115495.3	c.14319A>G	p.I4773M	4412	0.9	1.2	0	2	0.468	sporadic	AB839068

68	GPR98	NM_032119.3	NP_115495.3	c.18602A>C	p.N6201H	2030	2.2	1.2	0	2	1	AD/Mit, sporadic	AB839069
69	ISLR	NM_005545.3	NP_005536.1	c.146C>T	p.P49L	1505	0	2.1	0	2	0.914	AD	AB839070
70	KCNQ4	NM_004700.2	NP_004691.2	c.2014G>A	p.V672M	1809	0	1.4	0	1	0.984	sporadic	AB839071
71	KIAA1199	NM_018689.1	NP_061159.1	c.653G>A	p.R218H	2834	1.1	1.6	0	1	0.999	sporadic	AB839072
72	LHFP	NM_005780.2	NP_005771.1	c.299C>T	p.A100V	3282	0	1.2	0	1	1	sporadic	AB839073
73	LRP1	NM_002332.2	NP_002323.2	c.6493G>A	p.G2165R	304	0	1	0	1	1	AD/Mit	AB839074
74	LRP1	NM_002332.2	NP_002323.2	c.8801C>T	p.S2934L	284	1	0	0.8	1	0.999	sporadic	AB839075
75	LRP1	NM_002332.2	NP_002323.2	c.10802C>T	p.A3601V	263	1	0	0	1	0.702	sporadic	AB839076
76	LRP1	NM_002332.2	NP_002323.2	c.11780G>A	p.R3927H	1158	1.1	0	0	1	0.744	sporadic	AB839077
77	LRP1	NM_002332.2	NP_002323.2	c.12346C>T	p.H4116Y	2367	1.2	0	0	1	0.92	AR	AB839078
78	MARVELD2	NM_001038603.1	NP_001033692.1	c.166C>T	p.P56S	4151	1	0	0	1	0.999	sporadic	AB839079
79	MARVELD2	NM_001038603.1	NP_001033692.1	c.1115G>A	p.R372Q	4565	0	1	0	1	1	sporadic	AB839080
80	MARVELD2	NM_001038603.1	NP_001033692.1	c.1641T>A	p.D547E	1885	1.4	0	0	2	0.998	sporadic	AB839081
81	MYH14	NM_001077186.1	NP_001070654.1	c.2593A>C	p.K865Q	296	1	1.3	1.2	1	0.995	sporadic	AB839082
82	MYH14	NM_001077186.1	NP_001070654.1	c.4804G>A	p.E1602K	1165	0	1.2	0	1	0.966	sporadic	AB839083
83	MYH9	NM_002473.3	NP_002464.1	c.2404C>T	p.R802W	267	0	0.9	0	1	1	sporadic	AB839084
84	MYH9	NM_002473.3	NP_002464.1	c.4352C>T	p.A1451V	512	0.7	0	0	1	0.979	AD	AB839085
85	MYO15A	NM_016239.3	NP_057323.3	c.514C>T	p.L172F	710	1.1	0	0.9	1	1	AD/Mit	AB839086
86	MYO15A	NM_016239.3	NP_057323.3	c.554G>A	p.G185D	639	1.3	0	0	1	1	AD	AB839087
87	MYO15A	NM_016239.3	NP_057323.3	c.613T>C	p.F205L	1032	1.7	0	0	1	0.649	sporadic	AB839088
88	MYO15A	NM_016239.3	NP_057323.3	c.671A>G	p.Y224C	1254	0.9	0	0	1	0.999	sporadic	AB839089
89	MYO15A	NM_016239.3	NP_057323.3	c.4216G>A	p.E1406K	947	0	0.9	0	1	1	sporadic	AB839090
90	MYO15A	NM_016239.3	NP_057323.3	c.4322G>T	p.G1441V	1340	0	1.2	0	1	1	sporadic	AB839091
91	MYO15A	NM_016239.3	NP_057323.3	c.4828G>A	p.E1610K	426	0.9	0	0	1	1	AR	AB839092
92	MYO15A	NM_016239.3	NP_057323.3	c.4888C>G	p.R1630G	1381	0.9	0	0	1	1	sporadic	AB839093
93	MYO15A	NM_016239.3	NP_057323.3	c.5117G>T	p.G1706V	1643	1.8	0	0	1	1	sporadic	AB839094
94	MYO15A	NM_016239.3	NP_057323.3	c.9478C>T	p.L3160F	771	1.1	1.2	0	2	0.668	AD, sporadic	AB839095
95	MYO15A	NM_016239.3	NP_057323.3	c.9781A>T	p.N3261Y	577	0	0.7	0	1	0.997	sporadic	AB839096
96	MYO15A	NM_016239.3	NP_057323.3	c.10263C>G	p.I3421M	3233	2.9	3.7	1.1	4	0.931	sporadic	AB839097
97	MYO1A	NM_005379.2	NP_005370.1	c.2303G>A	p.R768Q	2265	0	0.9	0	1	0.996	sporadic	AB839098
98	MYO3A	NM_017433.4	NP_059129.3	c.426T>G	p.H142Q	3225	0	3.8	0	3	1	sporadic	AB839099
99	MYO3A	NM_017433.4	NP_059129.3	c.848A>C	p.Q283P	3696	1	0	0	1	0.758	sporadic	AB839100
100	MYO3A	NM_017433.4	NP_059129.3	c.1324C>A	p.H442N	2470	6.5	1.2	1	5	0.699	AD, AD/AR/Mit, sporadic	AB839101
101	MYO3A	NM_017433.4	NP_059129.3	c.1819G>T	p.V607F	4115	0.9	0	0	1	0.752	sporadic	AB839102
102	MYO6	NM_004999.3	NP_004990.3	c.614G>A	p.R205Q	1790	0	1.9	0	1	1	AD	AB839103
103	MYO6	NM_004999.3	NP_004990.3	c.3814C>T	p.R1272W	4492	1	0	0	1	1	sporadic	AB839104

104	MYO7A	NM_000260.2	NP_000251.2	c.2023C>T	p.R675C	739	1.1	1	0	2	1	AD, sporadic	AB839105
105	MYO7A	NM_000260.2	NP_000251.2	c.4806G>A	p.E1603K	1962	1	1	0	2	0.723	AD, sporadic	AB839106
106	MYO7A	NM_000260.2	NP_000251.2	c.6235C>T	p.R2079W	428	0.8	1.1	0	2	1	sporadic	AB839107
107	NDP	NM_000266.2	NP_000257.1	c.58G>A	p.G20R	3291	0	2.4	0	1	0.917	sporadic	AB839108
108	OTOF	NM_194248.1	NP_919224.1	c.157G>A	p.A53T	1034	3.2	1.3	1	3	0.476	sporadic	AB839109
109	OTOF	NM_194248.1	NP_919224.1	c.3683G>T	p.R1228L	529	1	0	0	1	0.765	sporadic	AB839110
110	OTOF	NM_194248.1	NP_919224.1	c.4417G>T	p.G1473C	1358	0	1.2	0	1	0.998	sporadic	AB839111
111	OTOF	NM_194248.1	NP_919224.1	c.5408A>C	p.E1803A	2050	3.9	0	0	1	0.627	AR	AB839112
112	PCDH15	NM_033056.3	NP_149045.3	c.298G>A	p.G100R	4003	0	1.3	0	1	1	AD/Mit	AB839113
113	PCDH15	NM_033056.3	NP_149045.3	c.833G>A	p.R278H	3135	1.1	0	0	1	0.997	sporadic	AB839114
114	PCDH15	NM_033056.3	NP_149045.3	c.944C>T	p.P315L	2799	1	0	0	1	1	sporadic	AB839115
115	PCDH15	NM_033056.3	NP_149045.3	c.2528C>A	p.A843D	3282	1.1	0	0	1	1	unknown	AB839116
116	PCDH15	NM_033056.3	NP_149045.3	c.2884C>T	p.R962C	2065	2.2	0	0	2	0.999	AR, sporadic	AB839117
117	PCDH15	NM_033056.3	NP_149045.3	c.3451G>A	p.G1151R	3773	1.9	0	1	2	1	sporadic	AB839118
118	POU4F3	NM_002700.1	NP_002691.1	c.736C>A	p.P246T	1444	0	0.9	0	1	1	sporadic	AB839119
119	RDX	NM_002906.3	NP_002897.1	c.869T>C	p.L290P	2435	0.9	0	0	1	1	unknown	AB839120
120	SLC17A8	NM_139319.1	NP_647480.1	c.1120G>T	p.A374S	3395	2.5	2.2	0	4	0.996	AD, sporadic	AB839121
121	SLC26A4	NM_000441.1	NP_000432.1	c.439A>G	p.M147V	1917	0.9	0	0	1	1	sporadic	AB839122
122	SLC26A4	NM_000441.1	NP_000432.1	c.1229C>T	p.T410M	2227	3.9	0	0	4	1	AR, sporadic	AB839123
123	SLC26A4	NM_000441.1	NP_000432.1	c.1579A>C	p.T527P	2141	0	1.4	0	1	0.999	sporadic	AB839124
124	SLC26A4	NM_000441.1	NP_000432.1	c.2168A>G	p.H723R	3943	10.9	2	0	13	1	AD, AD/Mit, AR, sporadic	AB839125
125	STRC	NM_153700.2	NP_714544.1	c.2914C>T	p.R972W	1109	0	0.8	0	1	1	sporadic	AB839126
126	STRC	NM_153700.2	NP_714544.1	c.4622G>A	p.R1541Q	2200	0	1	0	1	0.876	sporadic	AB839127
127	TCOF1	NM_001008656.1	NP_001008656.1	c.1045A>G	p.S349G	343	0	3.2	0	2	0.999	sporadic	AB839128
128	TCOF1	NM_001008656.1	NP_001008656.1	c.2344C>G	p.Q782E	1284	0.8	0	0	1	0.525	sporadic	AB839129
129	TCOF1	NM_001008656.1	NP_001008656.1	c.2666G>A	p.R889H	1860	0.8	0	0	1	0.999	AR	AB839130
130	TECTA	NM_005422.2	NP_005413.2	c.1471C>T	p.R491C	2792	3.5	0	0	2	1	sporadic, unknown	AB839131
131	TECTA	NM_005422.2	NP_005413.2	c.3511G>A	p.V1171M	2794	4	1.1	0.8	5	0.973	AD, sporadic	AB839132
132	TECTA	NM_005422.2	NP_005413.2	c.4198C>T	p.H1400Y	525	0	1.2	0	1	0.986	AD	AB839133
133	TECTA	NM_005422.2	NP_005413.2	c.4315C>A	p.L1439I	1815	0.9	0	0	1	0.999	AD/Mit	AB839134
134	TMPRSS3	NM_024022.1	NP_076927.1	c.212T>C	p.F71S	1256	0.8	1	0	2	0.932	sporadic	AB839135
135	TMPRSS3	NM_024022.1	NP_076927.1	c.280G>A	p.G94R	1497	0	1.9	0	2	0.999	sporadic, unknown	AB839136
136	TMPRSS3	NM_024022.1	NP_076927.1	c.316C>T	p.R106C	1127	0	1.1	0	2	1	AD/Mit, sporadic	AB839137
137	TMPRSS3	NM_024022.1	NP_076927.1	c.1159G>A	p.A387T	479	1.3	0	0	1	0.999	AR	AB839138
138	TRIOBP	NM_001039141.1	NP_001034230.1	c.154G>A	p.D52N	370	0	1.3	0	1	0.982	sporadic	AB839139
139	TRIOBP	NM_001039141.1	NP_001034230.1	c.4840G>T	p.G1614C	599	0.8	2.6	1.4	2	0.997	AD/Mit, AR	AB839140

140	TRIOBP	NM_001039141.1	NP_001034230.1	c.5519G>A	p.R1840H	1435	1	0	0	1	1	AR	AB839141
141	TRIOBP	NM_001039141.1	NP_001034230.1	c.6860G>A	p.R2287H	502	0.7	0	0	1	1	AR	AB839142
142	USH1C	NM_153676.2	NP_710142.1	c.188G>A	p.R63Q	536	2.7	0	0	1	1	unknown	AB839143
143	USH1C	NM_153676.2	NP_710142.1	c.2191C>T	p.R731W	1279	0	1.7	0	1	0.965	sporadic	AB839144
144	USH2A	NM_206933.1	NP_996816.1	c.206G>T	p.S69I	4565	0	0.9	0	1	0.977	sporadic	AB839145
145	USH2A	NM_206933.1	NP_996816.1	c.1678C>G	p.P560A	3609	0	1.1	0	1	0.884	sporadic	AB839146
146	USH2A	NM_206933.1	NP_996816.1	c.5608C>T	p.R1870W	3622	0.9	0	0	1	0.999	sporadic	AB839147
147	USH2A	NM_206933.1	NP_996816.1	c.1877G>A	p.R626Q	4554	1	0	0	1	1	sporadic	AB839148
148	USH2A	NM_206933.1	NP_996816.1	c.4070C>T	p.T1357M	3726	1.1	0	0	1	1	AR, sporadic	AB839149
149	USH2A	NM_206933.1	NP_996816.1	c.4616C>T	p.T1539I	2153	0.9	1.2	0	2	0.986	sporadic	AB839150
150	USH2A	NM_206933.1	NP_996816.1	c.7000A>G	p.N2334D	2667	1.2	0	0	1	0.954	AD/AR/Mit	AB839151
151	USH2A	NM_206933.1	NP_996816.1	c.7068T>G	p.N2356K	1900	2.2	3.4	0	5	0.454	AD/Mit, sporadic, unknown	AB839152
152	USH2A	NM_206933.1	NP_996816.1	c.9259G>A	p.V3087I	4209	0	2	0	1	0.816	AD	AB839153
153	USH2A	NM_206933.1	NP_996816.1	c.10852G>A	p.G3618S	3597	1.3	0	0	1	1	sporadic	AB839154
154	USH2A	NM_206933.1	NP_996816.1	c.10904C>A	p.T3635N	3759	1	0	0.9	1	0.681	AD/Mit	AB839155
155	USH2A	NM_206933.1	NP_996816.1	c.10999A>C	p.T3667P	2166	1.2	0	0	1	0.949	sporadic	AB839156
156	USH2A	NM_206933.1	NP_996816.1	c.14017T>C	p.Y4673H	4908	0	1.1	0	1	0.948	AR	AB839157
157	USH2A	NM_206933.1	NP_996816.1	c.15233C>G	p.P5078R	5165	1	1.3	0	1	1	AD	AB839158
158	USP11	NM_004651.3	NP_004642.2	c.484G>A	p.A162T	1610	1.3	0	0	1	0.885	sporadic	AB839159
159	WFS1	NM_006005.2	NP_005996.1	c.449C>T	p.A150V	794	1.1	0	0.7	1	0.964	AD/Mit	AB839160
160	WFS1	NM_006005.2	NP_005996.1	c.908T>C	p.L303P	2723	0	1.1	0	1	1	AD	AB839161
161	WNK2	NM_006648.3	NP_006639.3	c.947C>T	p.T316M	1606	0	1.1	0	1	1	sporadic	AB839162
162	WNK2	NM_006648.3	NP_006639.3	c.1703C>T	p.P568L	513	0	0.8	0	1	0.769	sporadic	AB839163
163	WNK2	NM_006648.3	NP_006639.3	c.5951C>T	p.P1984L	394	0.7	0	0	1	1	sporadic	AB839164
