# Supplementary material for: Targeted Exon Sequencing Successfully Discovers Rare Causative Genes and Clarifies the Molecular Epidemiology of Japanese Deafness Patients
Source: PLoS One. 2013 Aug 13;8(8):e71381. doi: 10.1371/journal.pone.0071381 (PMC3742761; doi:10.1371/journal.pone.0071381)
Supplement: Table S3 — Comparison of data between the current algorithm and VIPR. 93.5% (87/93) and 84.1% (37/44) of the mutations was detected in GJB2 and SLC26A4 genes already fully sequenced by Sanger sequencing, respectively. (PDF) [file pone.0071381.s005.pdf]

Supplementary Table S3. Comparison between the current algorithm and VipR

gene	mutation	mutations detected by Sanger sequencing	mutations detected by current MPS algorithm	mutations detected by VipR
GJB2	G45E; Y136X	10	10	10
	V37I	6	6	6
	T8M	1	1	1
	T86R	1	1	1
	R143W	3	3	0
	235delC	36	36	*
	35insG	1	1	*
	176_191 del16	3	3	*
	299_300delAT	5	5	*
	511insAACG	2	0	*
SLC26A4	H723R	13	13	13
	T410M	4	4	4
	919-2A>G	2	2	2
	M147V	1	1	0
	T527P	1	1	0
	S532I	1	0	0
	R581S	1	0	0
	2111insGCTGG	1	0	*
	1652insT	1	0	*
total		93	87	37

* VipR program could not pick up deletion/insertion mutation
